# Seasonal variation in diagnosis of invasive cutaneous melanoma in Eastern England and Scotland

**DOI:** 10.1016/j.canep.2015.06.006

**Published:** 2015-08

**Authors:** Fiona M. Walter, Gary A. Abel, Georgios Lyratzopoulos, Jane Melia, David Greenberg, David H. Brewster, Helen Butler, Pippa G. Corrie, Christine Campbell

**Affiliations:** a Department of Public Health & Primary Care, University of Cambridge, UK; b Health Behaviour Research Centre, University College London, UK; c Eastern Cancer Registry and Information Centre, Cambridge, UK; d Scottish Cancer Registry, NHS National Services Scotland, Edinburgh, UK; e University of Edinburgh, UK; f Cambridge University Hospitals NHS Foundation Trust (Addenbrooke's Hospital), UK

**Keywords:** Skin cancer, Melanoma, Seasonal variation

## Abstract

**Background:**

Worldwide, the incidence of cutaneous melanoma has been reported to be highest in the summer and lowest in the winter. Northern Irish data suggested seasonal variation for women only, especially those with thinner melanomas, sited on limbs. We interrogated two larger UK cancer registries for temporal differences in melanoma diagnosis and associated patient characteristics.

**Methods:**

Melanomas diagnosed from 2006 to 2010 in the Eastern England and Scottish cancer registries (*n* = 11,611) were analysed by month of diagnosis, patient demographics and melanoma characteristics, using descriptive and multivariate modelling methods.

**Results:**

More patients with melanoma were diagnosed in the summer months (June 9.9%, July 9.7%, August 9.8%) than the winter months (December 7.2%, January 7.2%, February 7.1%) and this pattern was consistent in both regions. There was evidence that the seasonal patterns varied by sex (*p* = 0.015), melanoma thickness (*p* = 0.002), body site (*p* = 0.006), and type (superficial spreading melanomas *p* = 0.005). The seasonal variation was greatest for diagnosis of melanomas occurring on the limbs.

**Conclusion:**

This study has confirmed seasonal variation in melanoma diagnosis in Eastern England and Scotland across almost all population demographics and melanoma characteristics studied, with higher numbers diagnosed in the summer months, particularly on the limbs. Seasonal patterns in skin awareness and related help-seeking are likely to be implicated. Targeted patient interventions to increase sun awareness and encourage year-long skin inspection are warranted.

## Highlights

• Two UK cancer registries examined for seasonal differences in melanoma diagnosis.
• More patients with melanoma were diagnosed in the summer than winter months.
• Seasonal patterns varied by sex, melanoma thickness, type and body site.
• Seasonal patterns were most marked for melanomas diagnosed on limbs.
• Awareness campaigns could highlight seasonal differences in melanoma diagnosis.

## 1 Introduction

Cutaneous melanoma is now the fifth commonest cancer diagnosed in the United Kingdom, following a rapid rise in incidence over the last few decades. While 11 cases per 100,000 population were diagnosed in 1999–2001, the age-standardised rate had increased by 55% to around 17 cases per 100,000 population in 2008–2010 [1]. The increase has been seen across sex and age groups, but most significantly among older men [2]. Importantly, more than a quarter of new melanoma cases were diagnosed in people aged less than 50 years in 2010, in contrast to only 11% among all cancers combined [1].

It is possible that some of the increase in incidence may be due to improved surveillance and earlier detection, as well as changes in diagnostic criteria [3]. However, most of the increasing incidence trends are considered to be due to exposure to ultra-violet radiation through increased frequency of sunbed use, intermittent unaccustomed exposure especially in childhood, and leisure-time exposure including holidays abroad and outdoor sport [4–6]. Approximately 86% of melanomas diagnosed in the UK in 2010 were estimated to be linked to exposure to ultra-violet radiation from the sun and sunbeds [7].

There is well documented seasonal variation in rates of melanoma diagnosis worldwide. Among light-skinned populations the incidence of melanoma is highest in summer and lowest in winter, irrespective of latitude [8,9]. However, detailed analysis of seasonal variation within the UK is limited to small datasets, and only data collected up until 2006: in the Oxford Region using data routinely collected between 1952 and 1975 [10], and in Northern Ireland where an analysis of data collected between 1984 and 2006 confirmed seasonal variation for women only, especially those with thinner melanomas and tumours diagnosed on the limbs [11].

We conducted an analysis of routinely collected clinical data from the Eastern England and Scottish cancer registries between 2006 and 2010, and compared diagnosis by month of the year, taking into account key factors including patient demographics as well as melanoma characteristics relevant to predicting disease outcome.

## 2 Materials and methods

### 2.1 Patient cohorts

We analysed routinely collected clinical data regarding invasive cutaneous melanoma registered by the National Cancer Registration Service- Eastern Office and the Scottish Cancer Registry between 1st January 2006 and 31st December 2010. While the regions have a comparable population size at approximately 5.7 and 5.2 million respectively, they are geographically distinct and have diverse socio-demographic characteristics. Primary sources of information included electronic and paper-based reports and clinical notes from hospitals and pathology laboratories. Recent reports have highlighted the completeness of recorded Breslow thickness for all melanomas, at more than 85% across both registries since 2006 (CI5 Vol X).

We abstracted data items related to patient, disease and temporal factors. Demographic variables included sex, age group at diagnosis (two groupings were used: <30 years, 30–49, 50–64 year and ≥65 years for comparison with crude incidence rates, and <50 years, 50–64 years, 65–74 years, ≥75 years for other analyses), and national quintile of small area measures of deprivation (using income domain of either the English or Scottish Index of Multiple Deprivation as applicable).

Disease data included melanoma Breslow thickness, histological type and body site of occurrence. Breslow thickness data were split into two categories: <2 mm, and ≥2 mm. Breslow thickness was reported in millimetres for 94% of the Scottish cases, and 89% of the Eastern England cases (17 Eastern England cases had Breslow thickness reported as group only). Histological types of melanomas were categorised using the ICDO(3) morphology codes: superficial spreading melanoma (SSM), nodular melanoma (NM), lentigo maligna melanoma (LMM), unclassified/unknown melanoma (UM) (codes 8720 NOS, 8723 NOS regressing), and others (OM) (codes 8722 Balloon cell, 8730 Amelanotic, 8740 Malignant melanoma in junctional naevus, 8741 Malignant melanoma in precancerous melanosis, 8744 Acral lentiginous, 8745 Desmoplastic, 8746 Mucosal lentiginous melanoma, 8761 Melanoma arising in congenital melancytic naevus, 8770 Mixed epithelial and spindle cell, 8771 Epithelioid cell melanoma, 8772 Spindle cell NOS, 8780 Blue naevus malignant). Anatomical site was coded using the International Classification of Diseases (ICD) 10 (four digit) as follows: C430 (lip), C431 (eyelid), C432 (ear), C433 (other and unspecified parts of face), and C434 (scalp and neck) were located on ‘Head and Neck’; C435 corresponded to ‘Trunk’; C436 to ‘Upper Limb’; C437 to ‘Lower Limb’; C438 (other specified sites of skin) and C439 (site unspecified) to ‘Other’ sites. Melanomas occurring in the eye or genital organs, and *in situ* melanomas were excluded.

Temporal differences were characterised by the month and year of melanoma diagnosis.

### 2.2 Statistical analysis

Initial descriptive analyses were undertaken to compare cases per year between the two regions, and to compare stratified Breslow thickness by patient demographics, tumour characteristics, and month of diagnosis. Multivariate analyses were then undertaken using a negative binomial regression to model the number of cases diagnosed in each month (outcome). The main exposure of interest was seasonal variation which was modelled using sine and cosine components with a period of one year. The model further included fixed effect exposures for sex, age, deprivation, region, Breslow thickness (as a binary variable <2 mm or ≥2 mm), site and histological type. Finally a longer term trend in incidence was accounted for with a cubic spline with 3 knots (a pragmatic choice providing more flexibility than a linear trend whilst remaining reasonably parsimonious). A negative binomial model was used as initial investigations suggested that more variation existed than suggested by the Poisson distribution (i.e. there was over-dispersion). As with Poisson regression, the negative binomial framework models counts rather than rates and so an offset equal to the log of the person time at risk needs to be included such that the outputs from the model may be interpreted as rate ratios. To do so we use the population at risk in each age by sex by deprivation group. This was calculated using 2008 national statistics aggregated up from the lower super output area level (a lower super output area is a geographic region defined for reporting of UK census data, each containing a population of around 1500 people). When initially specifying the model more complex parametrisations of seasonal variation were considered, however, early investigations suggested that higher order Fourier components did not significantly improve the fit of the model.

In addition to considering the overall seasonal trend we were also interested in whether the seasonal variation in incidence was dependant on other factors. In order to investigate this we considered interactions between the sine and cosine components and other variables retaining only those found to be statistically significant. Where more than one variable was found to have a significant interaction, interactions between those variables were also considered. As a supplementary analysis we repeated the final regression model treating Breslow thickness as a 4 category model (≤1 mm, 1–1.99 mm, 2–3.99 mm and ≥4 mm). Data analysis was undertaken using Stata 13 (Stata Corporation, College Station, TX).

## 3 Results

### 3.1 Sample description and incidence

A total of 11,611 invasive cutaneous melanoma cases were registered in both regions from 2006 to 2010: 5998 in Eastern England and 5613 in Scotland. The number of cases and crude incidence is shown in Table 1 by year, patient demographics and tumour characteristics. Analysis of overall melanoma incidence rates demonstrated a steady rise in numbers detected over the five years by 21% from 92 to 112 per 100,000 people per year. The incidence of melanomas was slightly higher in women than in men (109 versus 102 per 100 000), with higher incidence of diagnosed melanomas in the older age groups (<30 years 13 per 100,000 compared to 253 per 100,000 for >65 years). The incidence of melanoma decreased with increasing levels of deprivation (‘least deprived’ group 132 per 100,000 versus 71 per 100,000 in the ‘most deprived’ group). The majority (67%) of melanomas were relatively thin, and the commonest histological type was superficial spreading melanoma (54%). Slightly more melanomas were located on the trunk (29%) and lower limbs (27%) than on the upper limbs (21%) and head/neck areas (20%).

### 3.2 Seasonal variation in incidence

For the whole patient cohort, more melanomas were diagnosed in the summer months (June 9.9%, July 9.7%, August 9.8%) than the winter months (December 7.2%, January 7.2%, February 7.1%) and this pattern was consistent for both regions studied. Seasonal variation was evident when analysed by patient sex (particularly among women) and age (most marked in those aged less than 50 years) (Table 2). Seasonal variation was also evident when analysed by Breslow thickness, histological type and body site of occurrence (Table 3).

**Fig. 1:**
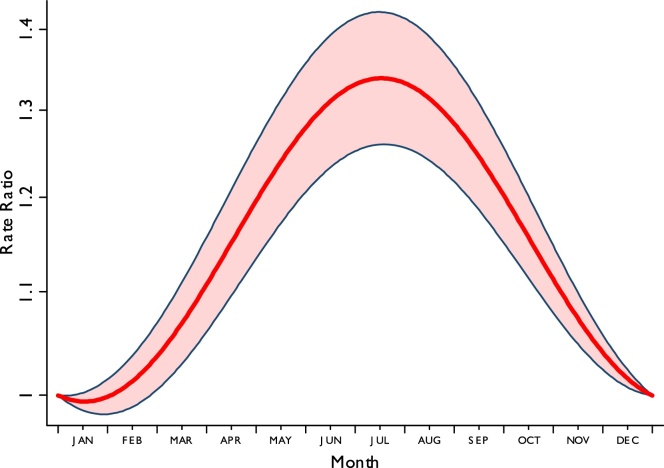
The average seasonal variability in melanoma incidence predicted from a model without interaction terms.

Multivariate analysis showed that after adjusting for patient and melanoma characteristics, there was very strong evidence (*p* < 0.001) of seasonal variability in melanoma diagnosis. The average seasonal variability predicted from a model without interaction terms is shown in Fig. 1 as rate ratios compared to 1st January each year. There was substantial variation in incidence over a year with rates over 30% higher in the summer months than the winter months and a peak incidence occurring in July (peak to peak rate ratio = 1.35 95% confidence interval (CI) 1.27, 1.43). This analysis excluded those patients with missing Breslow thickness, but similar patterns were seen in a model excluding tumour factors on the full data set, indicating no obvious bias by using a complete case analysis.

**Fig. 2:**
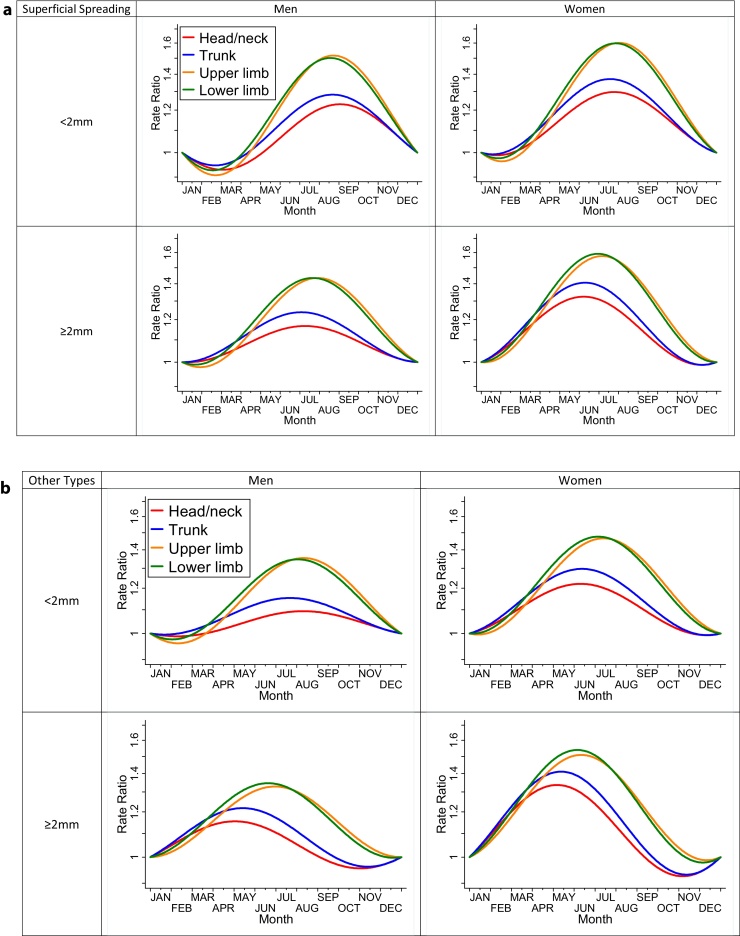
(a) Seasonal variability in Superficial Spreading Melanoma incidence by melanoma thickness, body site and patient sex predicted from a model including interaction terms (rate ratios compared to 1st January). (b) Seasonal variability in melanoma incidence for types other than Superficial Spreading Melanoma by melanoma thickness, body site and patient sex predicted from a model including interaction terms (rate ratios compared to 1st January).

In multivariate analysis using interaction terms we found no evidence that the seasonal variability was different for patients of different ages (*p* = 0.51), deprivation (*p* = 0.56) or region (*p* = 0.56). The seasonal variation by sex, Breslow thickness, body site and histological type is shown in Fig. 2; there is evidence of each being a moderating factor (*p* = 0.015 for sex, *p* = 0.002 for Breslow thickness, *p* = 0.006 for body site, and *p* = 0.005 for histological type). Seasonal variation varied by histological type: while it was distinct for superficial spreading melanoma (*p* = 0.005), there was no evidence of differential seasonal patterns for other types (*p* = 0.46). The final model also included two-way interactions between sex and body site, sex and melanoma thickness, and body site and thickness (*p* < 0.001 for all). Although “other/Unspecified” sites were included in the model we do not show the variation due to small numbers and large uncertainties.

The starkest finding illustrated in Fig. 2 is that the seasonal variation is around twice as large for melanomas arising on the limbs than those arising on the head, neck or trunk. There was evidence of a larger seasonal variation in women than in men. The difference between thinner and thicker melanomas was small, with the peak occurrence of thicker melanomas occurring slightly earlier than the summer months. Each line is plotted individually with confidence intervals in online Appendix 1. All of the 32 individual lines are statistically significant (*p* < 0.05) with the exception of two where the variation is weak (non-superficial spreading, thin melanomas, occurring in men on the head/neck (*p* = 0.44) or trunk (*p* = 0.29)). The trend for thicker melanomas to have a peak occurrence earlier in the year than thinner ones is also evident in the supplementary analysis splitting thickness into four categories (online Appendix 2).

## 4 Discussion

### 4.1 Main findings

This is the first study to compare and contrast seasonal variation in the diagnosis of cutaneous melanoma across English and Scottish populations, and it showed stronger seasonal variation in incidence than the previously reported Northern Irish data analysis. Seasonal variation was evident when analysed by sex, age, Breslow thickness, histological type and body site of occurrence. Seasonal variation was more marked in women and younger people in both regions, and was significantly greater on both the upper and lower limbs compared with the trunk and head/neck, in thinner (<2 mm) compared to thicker (≥2 mm) melanomas, and in superficial spreading melanomas compared with other histological types.

### 4.2 Comparison with existing literature

Our results contrast notably with those of Chaillol and colleagues [11], based on Northern Ireland Cancer Registry data, which only found seasonal variation in women with thin melanomas arising on the limbs. They did not observe seasonal variation in older men, thinner melanomas arising on the trunk, head or neck, or any thicker melanomas irrespective of age, sex and body site. The reasons for this contrast with our results are unclear, although the sample size of the Northern Irish study was five-fold smaller, therefore there may have not been enough power to detect such associations. The issue of power was further compounded by the stratified approach they took rather than the approach of using interactions that we have employed. Furthermore, our data were collected between 2006 and 2010 (compared with Challiol et al's analyses using data from cases diagnosed between 1984 and 2006), so that important differences in more recent patterns of UV exposure, social conventions regarding exposure of the skin, and help-seeking behaviour for skin changes in earlier decades, may also play a role.

Chaillol et al. also undertook a systematic literature review of studies examining whether seasonal variations were associated with sex, age, body site, histological type or Breslow thickness. They identified 15 studies (Europe (Norway, Germany, Greece, Italy, UK) 7, USA 5, Australia 2, Brazil 1) reporting data from 281 to 32,868 melanoma cases diagnosed between 1960 and 2006 [8–10,12–23]. Overall, they found that seasonal variations were more marked in women than men, in younger ages, and on the limbs more than the trunk or head and neck; our findings are strikingly similar. Seven studies reported greatest seasonal variation in superficial spreading melanomas, with three studies also reporting seasonal variation with lentigo maligna melanomas. Five studies found seasonal variation in thin lesions with none in thick lesions, while one Greek study found no seasonal variation with Breslow thickness. A more recent study by Keller and colleagues [24], set in Bavaria in Germany and analysing 11,901 malignant melanomas registered from 2003 to 2008 again shows contrasting results: in this population seasonal variation was most evident in thicker (≥2 mm) melanomas on the limbs. The only other previous study set in the UK reported the month of first attendance (rather than the month of histological diagnosis) of the 1019 melanomas registered by the Oxford Cancer Registry between 1952 and 1975 [10]. It showed peak months of presentation between July and September for men and women, most marked in people aged less than 55, both corresponding with our findings.

We report monthly figures showing pronounced seasonal variation, which may be influenced by a number of factors. UV exposure is likely to be important: not only are the limbs more likely to be visible in the summer months through wearing of short-sleeved tops, and shorts or skirts, but they are concomitantly more exposed to UV radiation. The causal effects of UV exposure on melanoma are well documented, as economic and social changes in recent years have contributed to increased sun exposure during holidays and outdoor sports, and the widespread use of indoor tanning [25–27]. While initiation of a melanoma seems unlikely to occur within a short interval following exposure, there is increasing evidence that short sun exposures can also promote rapid pathological changes in existing lesions which may result in visible changes, thereby contributing to seasonal variations in help-seeking and subsequent detection [24].

The visibility of the lesion is also likely to be a major contributing factor: lighter or less clothing worn on the limbs in the spring and summer months allows people to appraise their own skin more easily. It could have been hypothesised that enhanced visibility might lead to an ‘earlier harvesting’ of lesions that would have otherwise been diagnosed later in the year; however, the findings considering monthly variation by melanoma thickness (see Table 3, top super-row) indicate that thicker melanomas have a slightly earlier peak presentation than thinner ones. Visible lesions may also be appraised by others, particularly family members, friends and work colleagues, as well as opportunistically by healthcare professionals. Recent qualitative evidence suggests the importance of people's social network in prompting help-seeking for melanoma [28], while living alone is associated with longer patient delays in diagnosis with other cancers [29]. Although less marked than for the limbs, variation was observed for lesions on the trunk for both males and females, likely reflecting increased exposure during warmer weather or while on holiday. We found least seasonal variation in melanomas on the head and neck which is unsurprising given high visibility of these areas throughout the year. However, seasonal variation has also been described in countries where lightweight clothing is worn all year round; a Hawaiian study demonstrated seasonal variations for head and neck lesions, which would be expected to show less changes in visibility compared with lesions on the trunk and limbs [13]. This suggests that the impact of the visibility of the lesion on seasonal variations in melanoma diagnosis is complex, and other factors may also be important. Previous research from an Italian cohort has indicated an excess risk of melanoma and other skin cancer for people born in Spring or Autumn months [30], which nonetheless would not explain the patterns of excess risk in the summer months observed in our study.

### 4.3 Strengths and weaknesses of the study

The main strength of this study is that it used population-based data of high quality and completeness from two regions covering >10 million UK population. The most important prognostic factor, the Breslow thickness, was reported in millimetres for about 90% of the cohort. Furthermore, we were able to robustly account for potential confounding of seasonal variation by patient or tumour case-mix; in addition we examined the potential effect modification by the same factors.

This report has a number of limitations inherent in observational studies. Most importantly, the data are based on the accuracy of the information recorded at the National Cancer Registration Service and the Scottish Cancer Registry. We are not able to comment on time to presentation to the GP, or time from first seeing their GP to referral and histological diagnosis, as these data are not collected routinely by the registries – however it is highly unlikely that access to GPs and referral waiting times will exhibit such pronounced patterns of seasonal variation that would be necessary to explain the observed findings. We acknowledge that data from the two registries may not reflect national data, but suggest that the findings are unlikely to differ greatly in other UK regions.

### 4.4 Implications for practice and future research

Although there remains insufficient evidence for the introduction of national skin cancer screening programmes [31], a recent review suggested that melanoma early detection programmes aimed at high-risk individuals might be cost-effective [32]. Until then, raising awareness of skin cancer to promote timely diagnosis is vital [33]. Skin cancer awareness campaigns need not only to provide information on the early signs and symptoms that should prompt help-seeking (as well as encouragement to seek advice), but could capitalise on evidence of seasonal variation in diagnosis, which indicates that at least part of the population are more ‘skin-aware’ and show greater readiness for seeking medical help for suspected skin lesions during the summer months. This does not of course negate the need for ‘skin awareness’ throughout the year.

The beneficial role of friends and relatives in encouraging help-seeking behaviours in those who have a skin lesion has been documented [28,34]. There is evidence that such sanctioning of help seeking by others can be an important factor in reduced time to presentation [29,35], and again there is potential for skin cancer awareness campaigns to capitalise on this, particularly during the summer months. Similarly, there is scope for interventions among non-clinician professionals such as salon workers, chiropractors and others [36,37].

In conclusion, contemporary evidence from a large and geographically diverse population of UK patients with melanoma indicates notable seasonal variation in diagnosis, most likely demonstrating concordant seasonal variation in awareness of skin changes on self or others, and related help-seeking behaviour.

## Author contributions

The study was initiated by FMW and CC in the context of a Cancer Research UK/National Awareness and Early Diagnosis Initiative funded project reference: C8640/A12226: ‘Why do some patients present with thicker melanomas? A qualitative exploration of patients’ symptom detection, help-seeking decisions, and experiences of the pathways to diagnosis’. Methods were further developed and modified in discussions with GL, GAA and all other authors. GAA performed most analyses, with JM and HB. DG and DB oversaw the provision of data from the National Cancer Registry Service- Eastern Region and the Scottish Cancer Registry respectively. All authors commented on the methods and findings, and saw and approved the final draft of the paper.

## Ethics approval

Not required. An integral part of the Regional Cancer Registries was that anonymous data may be used for research purposes.

## Funding statement

The paper was materially supported by the National Institute for Health Research (NIHR-CS-012-030), supporting FMW through a Clinician Scientist award. The views expressed in this publication are those of the authors and not necessarily those of the NHS, the National Institute for Health Research or the Department of Health. During this project, GL was supported by a National Institute for Health Research post-doctoral fellowship (PDF-2011-04-047) to the end of 2014; and by a Cancer Research UK Clinician Scientist Fellowship award (A18180) from March 2015.

## Conflict of interests

The authors declare no conflicts of interest.

## Figures and Tables

**Table 1:** Sample description and crude incidence rates.

		*n*	%	Crude annual incidence per 100,000 population^a^	*p*-value
Total cases		11,611	100	21.2	

Year	2006	2020	17.4	18.4	<0.001
	2007	2142	18.4	19.5	
	2008	2499	21.5	22.8	
	2009	2500	21.5	22.8	
	2010	2450	21.1	22.3	

Region	East of England (total)	5998	51.7	21.0	0.226^b^
	2006	1024	8.8	17.9	
	2007	1043	9.0	18.2	
	2008	1314	11.3	23.0	
	2009	1308	11.3	22.9	
	2010	1309	11.3	22.9	
	Scotland (total)	5613	48.3	21.4	
	2006	996	8.6	19.0	
	2007	1099	9.5	21.0	
	2008	1185	10.2	22.6	
	2009	1192	10.3	22.7	
	2010	1141	9.8	21.8	

Sex	Men	5481	47.2	20.5	0.217
	Women	6130	52.8	21.9	

Age	<30	500	4.3	2.5	<0.001
	30–49	1756	15.1	15.4	
	50–64	4208	36.2	31.4	
	>65	5147	44.3	50.5	

Deprivation group	1 “least deprived”	3100	26.7	26.3	<0.001
	2	2866	24.7	23.0	
	3	2693	23.2	21.4	
	4	1885	16.2	18.0	
	5 “most deprived”	1067	9.2	14.2	

Breslow thickness	<2 mm	7787	67.1	14.2	<0.001
	≥2 mm	2868	24.7	5.2	
	Unknown	956	8.2	1.7	

Histological type	Superficial spreading	6221	53.6	11.3	<0.001
	Nodular melanoma	1355	11.7	2.5	
	Lentigo maligna melanoma	1145	9.9	2.1	
	Unclassified melanoma	2116	18.2	3.9	
	Others	774	6.7	1.4	

Anatomical site	Trunk	3373	29.1	6.2	<0.001
	Lower limb	3136	27.0	5.7	
	Upper limb	2482	21.4	4.5	
	Head and neck	2368	20.4	4.3	
	Unspecified and other sites	252	2.2	0.5	

a Incidence rates based on 2008 population figures.

b *p*-value for test of differences by region base on aggregate test of East of England versus Scotland only and does not consider annual variation.

**Table 2:** Distribution of melanoma incidence (%) by month of diagnosis and patient demographics (*n* = 11,611).

Month of diagnosis	Jan	Feb	Mar	Apr	May	Jun	Jul	Aug	Sept	Oct	Nov	Dec
Frequency (%)	834 (7.18)	829 (7.14)	918 (7.91)	849 (7.31)	979 (8.43)	1151 (9.91)	1129 (9.72)	1138 (9.80)	971 (8.36)	1036 (8.92)	940 (8.10)	837 (7.21)

Sex												
Men, %	390 (7.12)	396 (7.22)	436 (7.95)	382 (6.97)	452 (8.25)	529 (9.65)	502 (9.16)	555 (10.13)	441 (8.05)	489 (8.92)	465 (8.48)	444 (8.10)
Women, %	444 (7.24)	433 (7.06)	482 (7.86)	467 (7.62)	527 (8.60)	622 (10.15)	627 (10.23)	583 (9.51)	530 (8.65)	547 (8.92)	475 (7.75)	393 (6.41)

Age group												
<50 yrs, %	228 (7.26)	213 (6.79)	272 (8.67)	248 (7.90)	280 (8.92)	287 (9.14)	333 (10.61)	308 (9.81)	267 (8.51)	262 (8.35)	232 (7.39)	209 (6.66)
50–64 yrs %	227 (6.83)	254 (7.64)	265 (7.97)	241 (7.25)	259 (7.79)	339 (10.20)	314 (9.44)	342 (10.29)	289 (8.69)	295 (8.87)	263 (7.91)	237 (7.13)
65–74 yrs %	185 (7.82)	169 (7.14)	173 (7.31)	155 (6.55)	195 (8.24)	236 (9.97)	216 (9.13)	242 (10.22)	177 (7.48)	229 (9.67)	216 (9.13)	174 (7.35)
75+ yrs %	194 (6.98)	193 (6.94)	208 (7.48)	205 (7.37)	245 (8.81)	289 (10.40)	266 (9.57)	246 (8.85)	238 (8.56)	250 (8.99)	229 (8.24)	217 (7.81)

Region												
Scotland	402 (7.16)	400 (7.13)	459 (8.18)	404 (7.20)	487 (8.68)	559 (9.96)	505 (9.00)	550 (9.80)	472 (8.41)	494 (8.80)	476 (8.48)	405 (7.22)
Eastern England	432 (7.20)	429 (7.15)	459 (7.65)	445 (7.42)	492 (8.20)	592 (9.87)	624 (10.40)	588 (9.80)	499 (8.32)	542 (9.04)	464 (7.74)	432 (7.20)

Deprivation quintiles												
1 most affluent %	227 (7.32)	209 (6.94)	243 (7.84)	229 (7.39)	270 (8.71)	300 (9.68)	309 (9.97)	315 (10.16)	245 (7.90)	293 (9.45)	244 (7.89)	216 (6.97)
2%	206 (7.19)	213 (7.43)	228 (7.96)	225 (7.85)	226 (7.89)	300 (10.47)	272 (9.49)	269 (9.39)	231 (8.06)	239 (8.34)	254 (8.86)	203 (7.08)
3%	188 (6.98)	196 (7.28)	203 (7.54)	188 (6.98)	244 (9.06)	273 (10.14)	282 (10.47)	257 (9.54)	247 (9.17)	241 (8.95)	187 (6.94)	187 (6.94)
4%	140 (7.43)	135 (7.16)	145 (7.69)	144 (7.64)	162 (8.59)	180 (9.55)	165 (8.85)	187 (9.92)	156 (8.82)	168 (8.91)	159 (8.44)	144 (7.64)
5 most deprived %	73 (6.84)	76 (7,12)	99 (9.28)	63 (5.90)	77 (7.22)	98 (9.18)	101 (9.47)	110 (10.31)	92 (8.62)	95 (8.90)	96 (9.00)	87 (8.15)

**Table 3:** Distribution of melanoma incidence (%) by month of diagnosis and melanoma characteristics.

Month of diagnosis	Jan	Feb	Mar	Apr	May	Jun	Jul	Aug	Sept	Oct	Nov	Dec
Breslow thickness												
Total %	834 (7.18)	829 (7.14)	918 (7.91)	849 (7.31)	979 (8.43)	1151 (9.91)	1129 (9.72)	1138 (9.80)	971 (8.36)	1036 (8.92)	940 (8.10)	837 (7.21)
<2 mm %	547 (7.02)	536 (6.88)	590 (7.58)	572 (7.35)	644 (8.27)	765 (9.82)	746 (9.58)	790 (10.15)	690 (8.86)	703 (9.03)	642 (8.24)	562 (7.22)
≥2 mm %	216 (7.53)	217 (7.57)	246 (8.58)	207 (7.22)	275 (9.59)	290 (10.11)	288 (10.04)	258 (9.00)	209 (7.29)	242 (8.44)	231 (8.05)	189 (6.59)
Unknown %	71 (7.43)	76 (7.95)	82 (8.58)	70 (7.32)	60 (6.28)	96 (10.44)	95 (9.94)	90 (9.41)	72 (7.53)	91 (9.52)	67 (7.01)	86 (9.00)
Histological type												
Superficial spreading %	419 (6.74)	426 (6.85)	462 (7.43)	456 (7.33)	501 (8.05)	613 (9.85)	641 (10.03)	624 (10.03)	532 (8.55)	573 (9.21)	512 (8.23)	462 (7.43)
Nodular %	106 (7.82)	90 (6.64)	121 (8.93)	108 (7.97)	119 (8.78)	136 (10.04)	133 (9.82)	128 (9.45)	108 (7.97)	113 (8.34)	104 (7.68)	89 (6.57)
Lentigo maligna %	83 (7.25)	76 (6.64)	94 (8.21)	87 (7.60)	111 (9.69)	114 (9.96)	98 (8.56)	103 (9.0)	101 (8.82)	100 (8.73)	98 (8.56)	80 (6.99)
Unclassified/unknown %	161 (7.61)	171 (8.08)	176 (8.32)	156 (7.37)	182 (8.60)	210 (9.92)	196 (9.26)	208 (9.83)	162 (7.66)	195 (9.22)	159 (7.51)	140 (6.62)
Other %	65 (8.40)	66 (8.53)	65 (8.40)	42 (5.43)	66 (8.53)	78 (10.08)	61 (7.88)	75 (9.69)	68 (8.79)	55 (7.11)	67 (8.66)	66 (8.53)
Anatomical site^a^												
Head/neck %	185 (7.81)	186 (7.85)	179 (7.56)	188 (7.94)	207 (8.74)	216 (9.12)	214 (9.04)	222 (9.38)	194 (8.19)	194 (8.19)	201 (8.49)	182 (7.69)
Trunk %	257 (7.62)	256 (7.59)	270 (8.00)	238 (7.06)	275 (8.15)	341 (10.11)	314 (9.31)	322 (9.55)	270 (8.00)	296 (8.78)	270 (8.00)	264 (7.83)
Upper limb %	165 (6.65)	173 (6.97)	183 (7.37)	165 (6.65)	210 (8.46)	250 (10.07)	255 (10.27)	261 (10.52)	222 (8.94)	224 (9.02)	196 (7.90)	178 (7.17)
Lower limb %	205 (6.54)	187 (5.96)	260 (8.29)	233 (7.43)	271 (8.64)	328 (10.46)	322 (10.27)	316 (10.08)	269 (8.58)	296 (9.44)	259 (8.26)	190 (6.06)

a Unspecified and other sites *n* = 252 (Jan 22; Feb 27; Mar 26; Apr 25; May 16; Jun 16; Jul 24; Aug 17; Sep 16; Oct 26; Nov 14; Dec 23).
