# Supplementary material for: Seasonal variation in diagnosis of invasive cutaneous melanoma in Eastern England and Scotland
Source: Cancer Epidemiol. 2015 Aug;39(4):554–61. doi: 10.1016/j.canep.2015.06.006 (PMC4542219; doi:10.1016/j.canep.2015.06.006)
Supplement: Supplementary file 1 [file mmc1.docx]

**Online Appendix 1 - Seasonal variability in Melanoma incidence by melanoma type, melanoma thickness, body site and patient sex predicted from a model including interaction terms** (rate ratios compared to 1^st^ January). Each of these lines is shown in figure 2, here they are shown individually with 95% confidence intervals.

|  |  |
| --- | --- |
|  |  |
|  |  |
|  |  |
|  |  |
|  |  |
|  |  |
|  |  |
|  |  |
|  |  |
|  |  |
|  |  |
|  |  |
|  |  |
|  |  |
|  |  |

**Online Appendix 2a - Seasonal variability in Superficial Spreading Melanoma incidence by melanoma thickness, body site and patient sex predicted from a model including interaction terms** (rate ratios compared to 1st January).

| Superficial Spreading | Men | Women |
| --- | --- | --- |
| <1mm |  |  |
| 1-1.99mm |  |  |
| 2.3.99mm |  |  |
| ≥4mm |  |  |

**Online Appendix 2b - Seasonal variability in melanoma incidence for types other than Superficial Spreading Melanoma by melanoma thickness, body site and patient sex predicted from a model including interaction terms** (rate ratios compared to 1st January).

| Other Types | Men | Women |
| --- | --- | --- |
| <1mm |  |  |
| 1-1.99mm |  |  |
| 2.3.99mm |  |  |
| ≥4mm |  |  |
